# Effective recognition design in 8-ball billiards vision systems for training purposes based on Xception network modified by improved Chaos African Vulture Optimizer

**DOI:** 10.1038/s41598-024-63955-3

**Published:** 2024-06-17

**Authors:** WenKai Pan, Dong Zhu, Jutao Wang, Haiyan Zhu

**Affiliations:** 1 School of Information Science and Engineering, Linyi University, Linyi, 276000 Shandong China; 2 School of Physical Education and Health, Linyi University, Linyi, 276000 Shandong China

**Keywords:** Billiards, 8-ball, Billiards robot vision, Color image processing, Objective detection, Ball pattern recognition, Color space transformation, Thresholding, Position detection, Convolutional neural network, Xception network, Chaos African Vulture Optimization Algorithm, Engineering, Mathematics and computing

## Abstract

This research paper presents a comprehensive investigation into the utilization of color image processing technologies and deep learning algorithms in the development of a robot vision system specifically designed for 8-ball billiards. The sport of billiards, with its various games and ball arrangements, presents unique challenges for robotic vision systems. The proposed methodology addresses these challenges through two main components: object detection and ball pattern recognition. Initially, a robust algorithm is employed to detect the billiard balls using color space transformation and thresholding techniques. This is followed by determining the position of the billiard table through strategic cropping and isolation of the primary table area. The crucial phase involves the intricate task of recognizing ball patterns to differentiate between solid and striped balls. To achieve this, a modified convolutional neural network is utilized, leveraging the Xception network optimized by an innovative algorithm known as the Improved Chaos African Vulture Optimization (ICAVO) algorithm. The ICAVO algorithm enhances the Xception network's performance by efficiently exploring the solution space and avoiding local optima. The results of this study demonstrate a significant enhancement in recognition accuracy, with the Xception/ICAVO model achieving remarkable recognition rates for both solid and striped balls. This paves the way for the development of more sophisticated and efficient billiards robots. The implications of this research extend beyond 8-ball billiards, highlighting the potential for advanced robotic vision systems in various applications. The successful integration of color image processing, deep learning, and optimization algorithms shows the effectiveness of the proposed methodology. This research has far-reaching implications that go beyond just billiards. The cutting-edge robotic vision technology can be utilized for detecting and tracking objects in different sectors, transforming industrial automation and surveillance setups. By combining color image processing, deep learning, and optimization algorithms, the system proves its effectiveness and flexibility. The innovative approach sets the stage for creating advanced and productive robotic vision systems in various industries.

## Introduction

Billiards, among all ball sports, stands out due to the variety of games it offers, primarily because of the numerous balls involved. Listing all the different variations of billiards played worldwide is a challenging endeavor^1^. The sport is typically categorized into three main branches: Pocket billiards (pool), Snooker, and Carom billiards (carom). Pocket billiards, also known as pool, is the most popular style globally, attracting enthusiasts of all skill levels who enjoy playing various games within this category during their leisure time. This style involves 15 balls, including colored and single-colored balls, along with a white cue ball^2^. Billiard tables are available in three standard sizes: 7, 8, and 9 feet. Depending on the specific game being played, all the balls or a subset of them may be used. Common pocket billiard games such as 8-ball, 9-ball, and 10-ball differ not only in the number of balls used but also in rules, layout, and strategies required to emerge victorious^3^.

Meanwhile, the game of 8-ball has gained widespread recognition among the general public, even among those who have never played billiards before. This particular game is played on pocket billiard tables and involves the use of 15 target balls, which can be either of a single color or a combination of two colors, along with a white ball known as the cue ball.

In the game of 8-ball, balls numbered 1 to 7 share a uniform color, while balls numbered 9 to 15 exhibit a combination of two colors. The ball numbered 8 holds a special significance as it represents the final ball in the game. The arrangement of the balls follows a triangular pattern, with the crucial requirement that the number 8-ball must be positioned at the center of the triangle. Prior to the commencement of the game, the balls are carefully arranged at the bottom of the table and are subsequently broken and set into motion with the aid of a triangle.

Once the break has occurred, each player must select a specific group of balls to play with^4^. The first ball that a player successfully pockets determine their designated group for the remainder of the game. In order to emerge victorious, a player must pocket all the balls within their assigned group before attempting to pocket the number 8-ball^5^. It is important to note that when a player has successfully pocketed all their designated balls, they must then proceed to legally and accurately pocket the number 8 ball in order to secure their win.

Sensors are essential components in a vision system, as they are responsible for detecting and identifying objects^6^. Usually, cameras are employed as the main sensors to capture images of the table from different perspectives. Subsequently, computer vision algorithms are utilized to analyze the images and recognize the billiard balls and other game elements.

The development of effective recognition design in vision systems tailored for 8-ball billiards is a significant advancement in this field^7^. These systems are designed to enhance the training process by offering real-time feedback, strategic insights, and detailed analytics of gameplay.

The use of vision systems in sports has gained increasing importance due to their ability to capture and analyze high-speed actions that are often imperceptible to the human eye. In the case of 8-ball billiards, these systems can track the movement of balls, detect shot angles, and accurately predict the outcomes of strikes. This not only helps players improve their skills but also provides coaches with a valuable tool to assess performance and develop targeted training programs.

The integration of vision systems into sports training aligns with the broader trend of utilizing technology to enhance athletic performance^8^. This highlights the significance of visual acuity and processing in sports performance. In the context of billiards, where precision and strategy play a crucial role, the presence of an effective vision system becomes even more vital.

Different research work was proposed in the field of using vision systems in billiards. For example, Zhang et al. presented a novel application of image processing technology aimed at assisting novice billiard players through the implementation of an assisted hitting system^9^. This system incorporated several techniques such as double-peak histogram threshold segmentation, edge detection, Hough transform for ball belt and billiard ball detection, and a neural network for identifying the white ball specifically. Additionally, it provided shot suggestions based on dynamic formulas that consider the force and angle required for hitting, while also simulating the trajectory of the ball against the background. The conducted experiments successfully demonstrated the system's efficacy in accurately recognizing objects and simulating paths, thus offering significant support for beginners in enhancing their billiards skills. However, it is important to acknowledge certain limitations of this work, including its potential reliance on the quality and angle of input images, its performance in varying lighting conditions, and the adaptability of the suggested hitting strategies to individual players' styles and skill levels, which have not been extensively explored.

Park et al. proposed the Intelligent Carom Billiards Assistive System (ICBAS) as a newly developed tool to assist novice Carom Billiards players^10^. Monocular vision was utilized to detect the configuration of the cue ball and automatically calculates and was displayed solution paths for the player. By incorporating the widely used five and a half system for path calculation and considering the real-time prediction of cue ball paths influenced by the cue stick's direction, the system projects these paths onto the pool table for easy comparison and execution by the player. To ensure accurate path calculations and predictions, the system employed principal component analysis for the detection of ball movement to enhance the system's robustness. Also, the system continuously recalculates and updates the paths whenever the balls stop moving, providing continuous assistance to the player. To validate the practicality of the system, experiments were conducted using a real pool table. These experiments were demonstrated the system's effectiveness in assisting players during gameplay. However, it was important to acknowledge certain limitations of the system. Firstly, the system relied on specific environmental conditions for accurate vision detection, which may pose challenges in different playing environments. Secondly, the system encountered difficulties in adapting to different styles of play or unusual table setups. Lastly, players need to interpret and adjust to the guidance provided by the system, and its effectiveness may vary from player to player. By automatically calculating and displaying solution paths based on the cue ball's configuration, this system provides valuable assistance during gameplay. While there are limitations to consider, the system's practicality has been demonstrated through experiments conducted on a real pool table.

Zhang et al. collected a distinctive dataset encompassing break shot layouts, ball trajectories, and detailed performance statistics^11^. The scarcity of such data was attributed to the sport's underrepresentation in current studies, which predominantly focus on more popular sports like football and basketball. By utilizing this dataset, the researchers delved into various analytical tasks such as predicting and generating layouts, along with conducting similarity searches on trajectory data. The ultimate goal was to provide benefits to coaches, players, and fans. Through a series of extensive experiments, the study showcased the effectiveness of the proposed methods, highlighting their potential to enhance understanding and strategies in the realm of billiards. However, potential limitations of this work may include biases in data collection, the challenge of generalizing findings across different playing styles or conditions, and the reliance on the quality and comprehensiveness of the dataset. It is acknowledged that the dataset may not capture all nuances of the game or be fully representative of the global billiards playing community.

Sousa et al. presented an augmented reality tool designed to enhance the gaming experience for inexperienced or novice snooker players by offering real-time game assistance^12^. Through the utilization of a setup comprising a projector and a Kinect 2 sensor positioned above the snooker table, the system captures depth data and recognizes crucial elements such as the table's rails, the balls' positions, the cue's orientation, and the balls' interactions. Subsequently, it computed and anticipated the paths of the balls after being struck. These anticipated paths, in conjunction with visual enhancements and menus, were displayed directly onto the table, furnishing players with immediate visual cues. The system touted an impressive shot prediction accuracy rate of 98%, assuming there are no ball bounces. Nevertheless, the drawbacks of this study is center on its reliance on the accuracy and calibration of the sensor and projector configuration, potential difficulties in precisely forecasting outcomes in more intricate shots involving bounces, and the system's adaptability in diverse lighting conditions or settings that could impact sensor precision.

Gao et al. explored the progress in robot vision systems for 8-ball billiards, introducing an effective multi-objective recognition method that covers both the detection and pattern recognition of billiard balls^13^. The method begun by dividing the process into background and foreground separation using normalized RGB color space and histogram statistics for image pre-processing. To accurately identify and separate individual balls, it combines an enhanced Hough Transform (HT) algorithm with the Least Squares (LS) method, improving noise reduction and circle center fitting precision. This approach enables the precise localization of each ball on the table through a multi-ball detection strategy, achieving a remarkable detection accuracy of 99.4% in an average time of 0.65 s, surpassing conventional techniques like the Circular Hough Transform and K-means clustering. Additionally, the study introduces the application of Convolutional Neural Networks (CNN) for the intricate task of pattern recognition, distinguishing between solid and striped balls. The CNN model's training effectiveness is enhanced through color segmentation, morphological operations, image rotation for dataset expansion, and a pre-training phase for weight matrix optimization, resulting in a recognition rate exceeding 98.5%, which outperforms traditional methods. Nevertheless, potential limitations of this research may involve the reliance on specific lighting conditions for optimal image processing, the computational requirements of the proposed algorithms, especially in real-time scenarios, and the difficulty of generalizing the system's performance across various billiards table configurations or variations in ball appearance beyond standardized test conditions.

The current review of literature highlights a few gaps in research and outlines the goals of the proposed work. Existing billiards vision systems often face challenges with different lighting, table setups, and playing styles. The proposed system aims to improve adaptability by using strong color image processing techniques and a flexible deep learning model. By addressing variations in ball appearance, the methodology ensures accurate ball detection and pattern recognition across various billiard games, regardless of the ball colors and patterns. A main objective is to offer personalized assistance to players, providing real-time feedback and strategic insights that consider their individual playing styles and skill levels, thus enhancing their training experience. Additionally, the current literature lacks a comprehensive solution for efficient and accurate ball detection and pattern recognition. The proposed methodology fills this gap by utilizing a modified convolutional neural network optimized by the ICAVO algorithm, significantly enhancing the system's precision and reliability in these crucial tasks.

The reason behind this proposed work stems from the gaps and limitations found in the current state of robotic vision systems for billiards. While existing systems have made important contributions, they often struggle with different lighting, table setups, or playing styles, relying on specific environmental conditions. What is needed is a vision system that can adapt and handle various playing environments and ball appearances. Moreover, the current literature lacks a comprehensive solution for accurate and efficient ball detection and pattern recognition, which are crucial for a reliable system performance. This research aims to address these gaps and improve the overall effectiveness of robotic vision systems in the realm of 8-ball billiards and beyond.

This study investigates the development and execution of a vision system tailored for 8-ball billiards practice. It discusses the difficulties associated with identifying intricate patterns, the significance of immediate feedback, and the influence of these systems on player progress. Through an analysis of the existing technological landscape and its utilization in billiards practice, the research seeks to add to the continuous conversation on sports vision systems and their capacity to transform training approaches.

The methodology of the study consists of two main phases: the first one involves detecting balls using the vision system, while the second one focuses on recognizing patterns to differentiate between solid and striped billiard balls. The vision system plays a crucial role in capturing detailed images of the billiard table, which helps in accurately determining the positions and characteristics of the balls. After the detection of the balls, an advanced pattern recognition algorithm is utilized to examine the color patterns and textures of the balls, with a specific emphasis on distinguishing between solid and striped balls. To achieve this, deep learning techniques are utilized to create precise mathematical models for each type of ball, which aids in a smooth and dependable classification process.

## BALL detection

### The vision system for 8-ball image aquation

**Figure 1:**
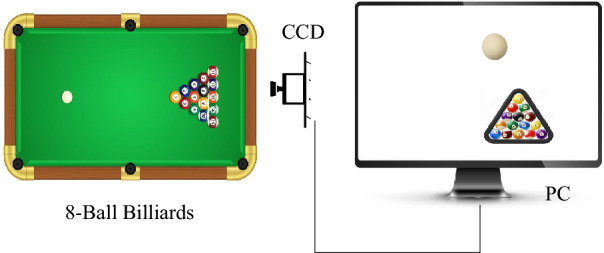
The vision system for 8-ball image aquation.

The visual system created for the 8-ball billiards robot includes key elements like the billiards table, individual balls, a high-resolution CCD camera, and a laptop for data processing, as shown in Fig. 1A. In order to analyze and track ball movements efficiently, the CCD camera is strategically positioned directly above the billiards table. Through precise adjustments and calibration settings, the camera captures a detailed 2D top-down view of the entire playing surface, as illustrated in Fig. 1B.

This top-down perspective provides a clear and unobstructed view of the ball positions and interactions, making it easier to track and analyze the game dynamics in real-time. This setup ensures that the robot's visual system can effectively monitor and interpret the gameplay, allowing it to make strategic decisions and accurate shots during the billiards match.

### Rectification of image distortion

In the realm of 8-ball billiards vision systems, it is imperative to rectify image distortion to ensure precise ball and table identification. Image distortion can greatly impact the perceived location and form of objects in the image, resulting in inaccuracies in the system's decision-making process. To tackle this issue, a two-step method can be implemented: distortion detection and distortion correction.

#### Detection of distortion

The initial step involves recognizing the type and degree of distortion present in the images captured by the vision system. Common forms of distortion include radial distortion, which causes straight lines to appear curved, and tangential distortion, which distorts the image. By examining the geometric discrepancies in the images, we can measure the distortion parameters.

#### Correction of distortion

Once the distortion parameters are determined, a correction algorithm can be applied. This study is to utilize the camera model to calculate the camera's intrinsic and extrinsic parameters. With these parameters, the distorted image points are accurately realigned to their correct positions using mathematical models like the Brown-Conrady model or the polynomial model.

The Brown-Conrady model, for example, addresses radial and tangential distortion by utilizing the subsequent equations:

1 $${x}_{corrected}=x(1+{k}_{1}{r}^{2}+{k}_{2}{r}^{4}+{k}_{3}{r}^{6})+2{p}_{1}xy+{p}_{2}({r}^{2}+2{x}^{2})$$

2 $${y}_{corrected}=y(1+{k}_{1}{r}^{2}+{k}_{2}{r}^{4}+{k}_{3}{r}^{6})+{p}_{1}({r}^{2}+2{y}^{2})+2{p}_{2}xy$$

In the case where ($x$) and ($y$) represent the coordinates of a point within the distorted image, ($r$) indicates the distance from the point to the center of the image, while (${k}_{1}$, ${k}_{2}$) and (${k}_{3}$) stand for the radial distortion coefficients, and (${p}_{1}$) and (${p}_{2}$) denote the tangential distortion coefficients.

**Figure 2:**
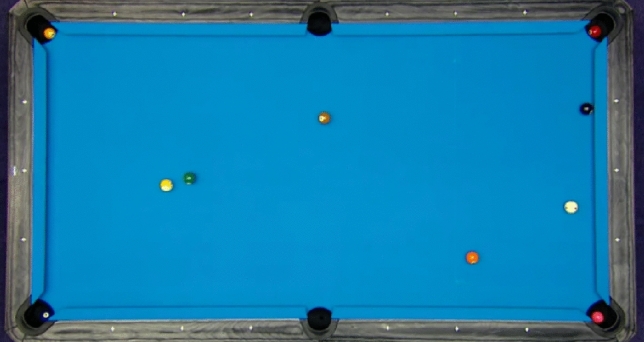
A sample of distortion corrected image.

By implementing these adjustments, we can acquire an unaltered depiction that precisely portrays the scenario, facilitating efficient identification and examination in the 8-ball billiards visual system. Executing such a rectification approach necessitates meticulous calibration of the camera system and potentially incorporates machine learning methods to enhance the precision of the distortion model. The ultimate objective is to attain a visual system that can consistently interpret the condition of the billiards table and the locations of the balls, enabling advanced capabilities such as automated scoring or augmented reality overlays for training and strategy planning. The corrected image is illustrated in Fig. 2.

By implementing the distortion correction technique, it is evident that the visual data's quality is greatly improved. This guarantees that subsequent algorithms for image processing and analysis can operate with greater efficiency, resulting in enhanced performance in tasks like object detection, tracking, and recognition. In the specific case of the 8-ball billiards robot, distortion-corrected images play a vital role in accurately identifying the positions of the balls on the table, predicting their paths, and making accurate decisions for gameplay.

### Billiard bag detection

A series of complex procedures has been meticulously established to precisely detect the position of the billiard ball. These procedures involve a variety of advanced techniques, such as color space transformation and mathematical operations, which aim to extract accurate spatial information from the visual data. Color space transformation plays a crucial role in this process by converting the captured RGB values into alternative HSV color space.

This allows the system to effectively isolate and distinguish the unique color of the billiard ball from its surroundings, facilitating reliable identification and localization. Additionally, mathematical operations are applied to the transformed color data to define the boundaries and contours of the billiard ball in the image. This includes utilizing sophisticated algorithms for edge detection, thresholding, and morphological operations, enabling the system to precisely determine the spatial extent and position of the billiard ball within the visual frame.

By integrating these intricate procedures, the vision system can accurately determine the exact location of the billiard ball, providing a solid foundation for subsequent analysis and decision-making processes in the context of the 8-ball billiards robot. The meticulous implementation of these procedures highlights the system's ability to effectively process visual data and extract crucial spatial information for seamless gameplay analysis and strategic decision-making.

At first, the color space transformation has been applied. Algorithm 1 shows the method of the converting the RGB color space into the HSV.

**Figure:**
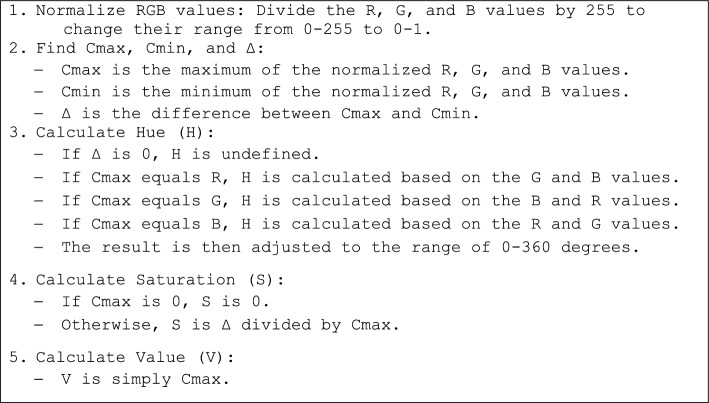
Algorithm 1 The method of the converting the RGB color space into the HSV.

**Figure 3:**
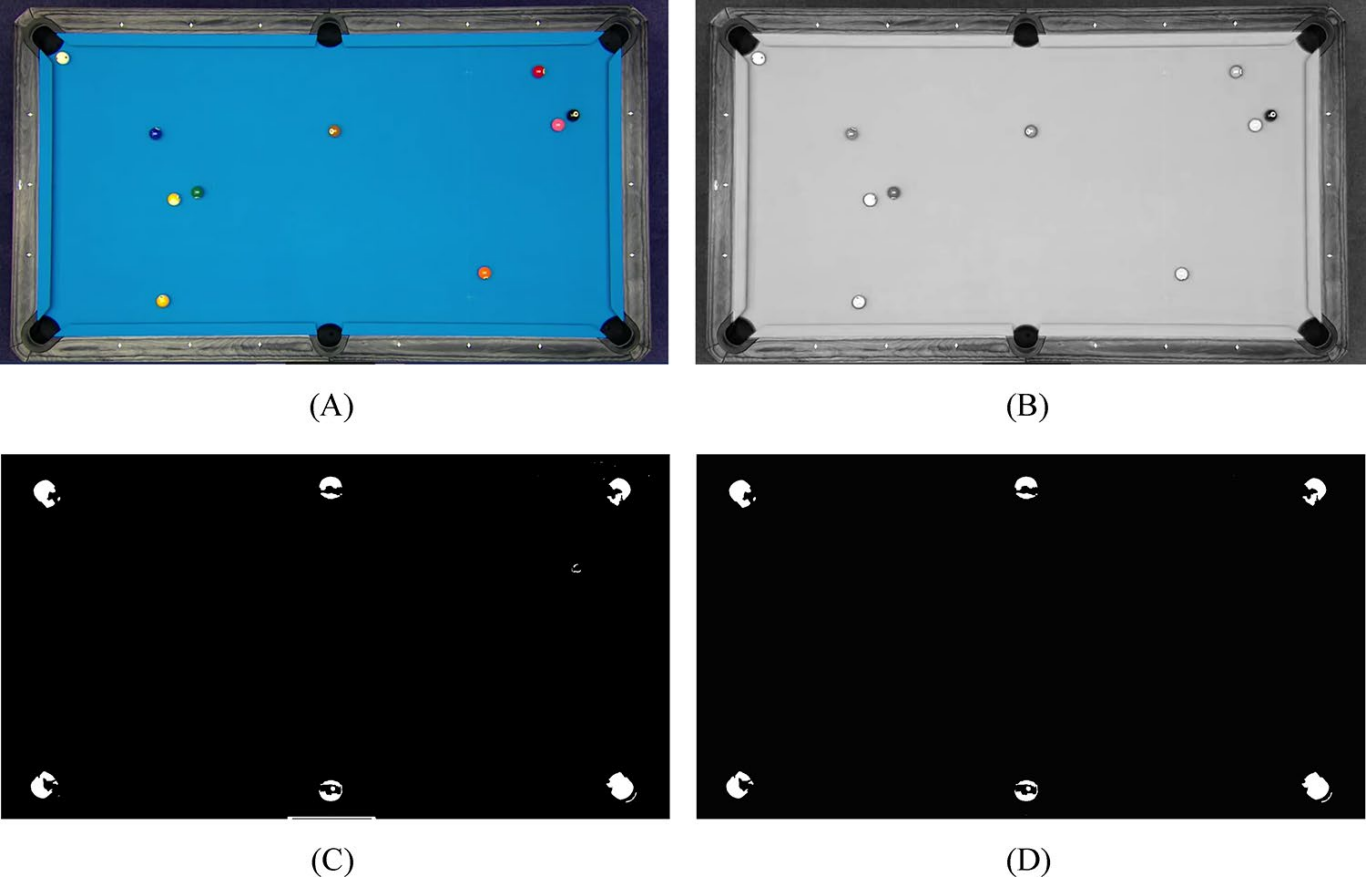
Billiard bag detection: (**A**) input RGB image, (**B**) “V” channel, (**C**) “V < 0.0830”, and (**D**) labeling and removing the fine areas.

In this study, the “value” color space is used for this stage. Based on the trials and errors and based on the table color, the “Value” channel with less than 0.0830 is selected to determine the Billiard bags (VV). Afterward, a structuring element in the shape of a disk with a radius of 20 has been considered an is used for mathematical morphology including closing to the binary image VV using this structuring element, resulting in the creation of a mask. Afterward, proceed to fill any hole’s present in the binary image. Finally, execute the labeling process and subsequently remove any small areas detected within the image. Figure 3 shows the applying this conversion to a sample image.

The pseudocode of this stage is given in the following:

**Figure:**
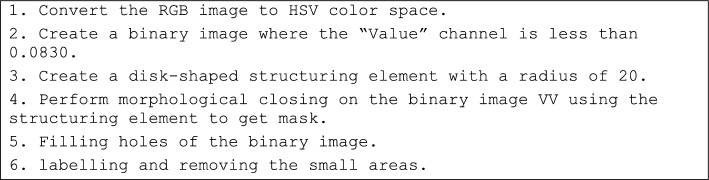
Algorithm 2 Billiard bag detection.

### Position detection of billiard table

Subsequent to the initial steps, where the billiard bags were recognized and their positions determined, the subsequent stage involves choosing the most extensive area among these six bags. This selection procedure concentrates on isolating the most notable and significant region on the billiards table, usually corresponding to a cluster of balls in close proximity or a concentrated group that stands out in terms of size or arrangement.

Once this primary area is pinpointed, a meticulous cropping operation is carried out to widen the visual scope for further analysis. Specifically, a region encompassing twice the dimensions of the largest bag area is cropped from all four directions of the table. This expanded cropping approach ensures that sufficient surrounding space is encompassed in the captured image, providing a broader context for comprehending the spatial relationships and configurations within the vicinity of the selected bag area.

By extending the cropped region in all directions around the largest bag area, the system acquires a more comprehensive view of the billiards table layout, facilitating a thorough examination of the surrounding elements and potential interactions. This enhanced perspective supports advanced analysis techniques and decision-making processes, enhancing the overall efficiency and accuracy of the vision system in interpreting and responding to the dynamics of the billiards game.

The summarized steps for position detection of billiard table are as follows. As can be observed, at first, the main table with color space cb < 165, where $cb$ can be achieved as follows:

3 $$Cb=128-37.945\times \frac{R}{256}\times 74.494\times \frac{G}{256}+112.439\times \frac{B}{256}$$

**Figure 4:**
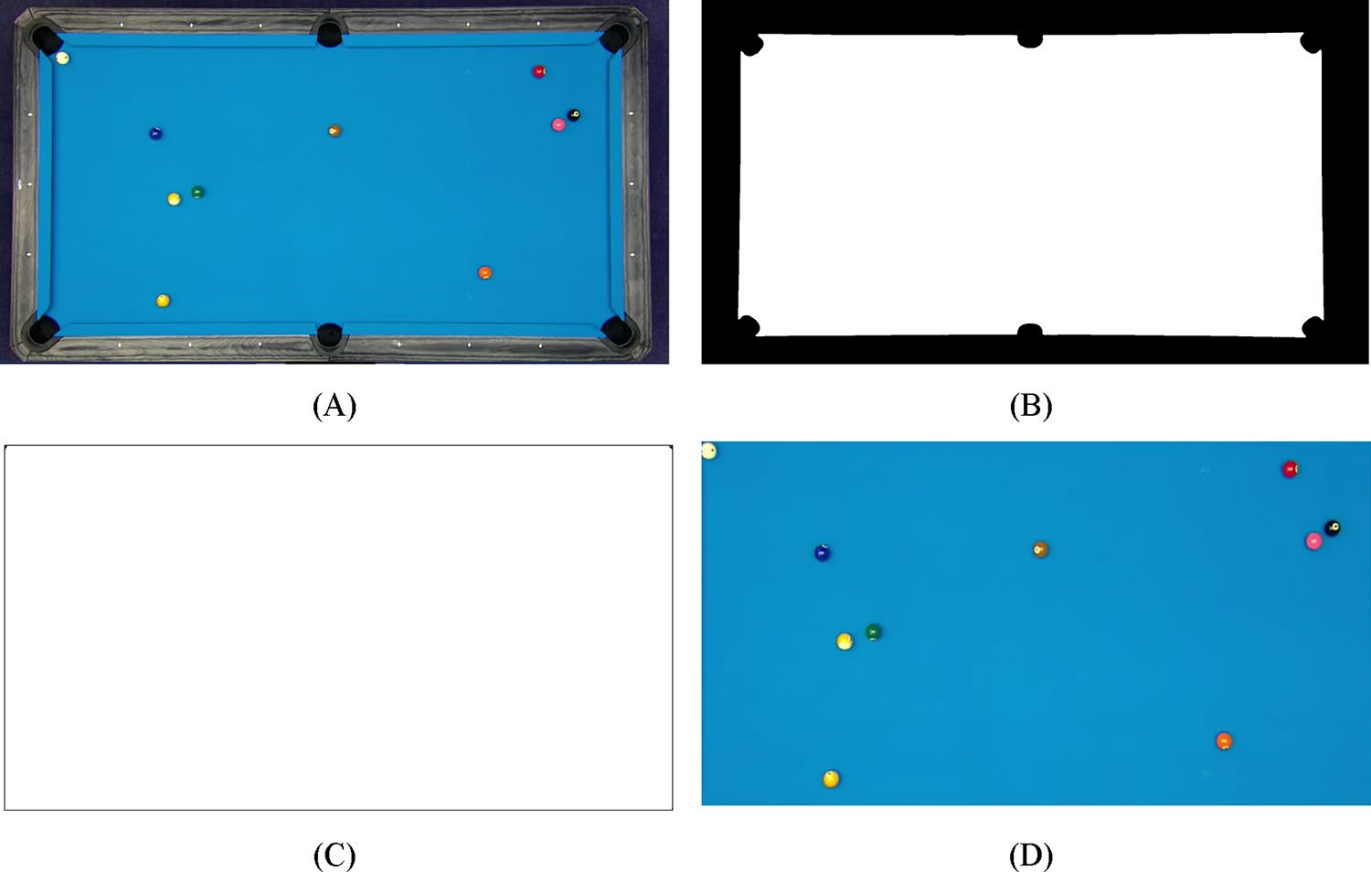
Position detection of billiard table: (**A**) input image, (**B**) image after table detection, (**C**) cropping the extra parts, (**D**) cropping the same similar sizes of (**D**) from (**A**).

Subsequently, morphological closing and hole-filling techniques are employed to refine the surface of the billiard table. Following this refinement, a cropping process guided by the aforementioned model is implemented. As a result, utilizing the specified cropping dimensions, the final table is accurately identified and delineated. Figure 4 shows the position detection of billiard table.

**Figure 5:**
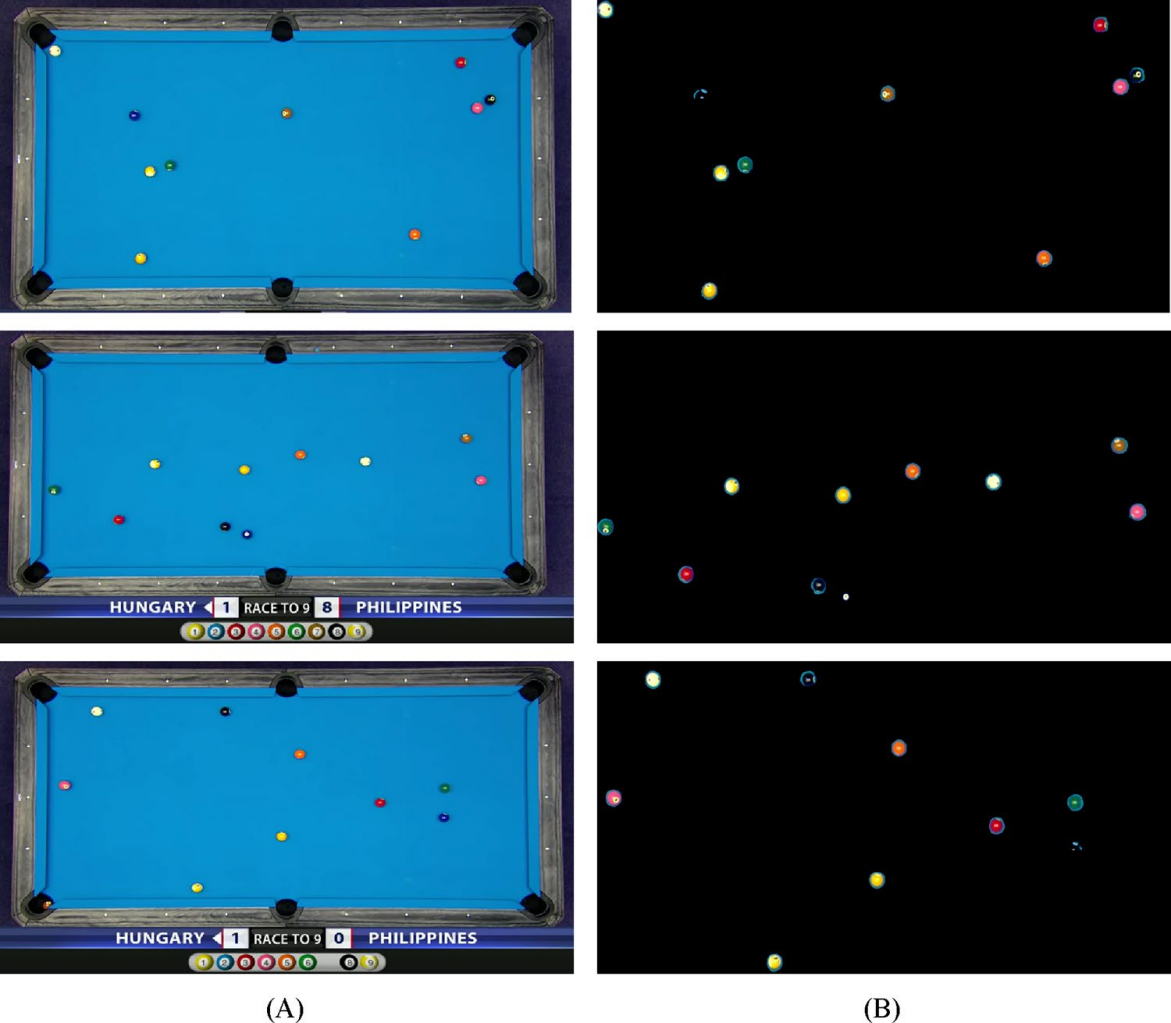
Shows some sample examples of final balls segmentation: (**A**) original image, (**B**) image after balls segmentation.

In general, the series of image processing steps illustrates the methodical approach employed to precisely identify and separate the billiard table from the input image. This, in turn, facilitates additional analysis and applications like object tracking, measurements, and improved visualization. Figure 5 shows some sample examples of final balls segmentation based on the proposed method.

## Pattern recognition of balls

**Figure 6:**
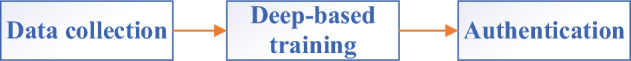
The steps involved in the recognition process.

In this study, we aim to employ a vision system-based method for the detection of balls on the billiard table. Subsequently, pattern recognition is applied to differentiate between solid and striped balls. To the human eye, solid balls appear as a single color, whereas striped balls exhibit a mix of colors, which can lead to potential misidentification. This research utilizes a machine vision system and deep learning to create an accurate mathematical model of these two types of balls, facilitating their easy detection. Figure 6 illustrates the steps involved in the recognition process.

### Data collection

In this study, we present a new method for detecting multiple balls. This method involves segmenting and categorizing ball images based on their patterns (striped/solid) in various gameplay scenarios. To ensure consistency, the segmented ball images are resized to a uniform dimension of 12 × 12 pixels. The initial dataset consists of 500 images, divided into a training set of 400 images (200 striped balls and 200 solid balls) and a test set of 100 images.

Since the main difference between solid and striped balls lies in the pattern of stripes, we acknowledge that color images contain unnecessary information that could prolong the training process and reduce the effectiveness of the deep network. To address this, we employ a preprocessing step that converts color images to grayscale through color segmentation. This approach is expected to streamline the training process and improve the accuracy of the model in distinguishing between the two types of balls.

### Convolutional neural network

This network has been found to be an efficacious approach for recognizing the patterns. In general, there are various layers within deep convolutional neural network, such as an entirely linked layer, pooling layers, and convolutional layers. The local trained filter has been employed to extract some visual data through input data in the present organization. The step of pooling might reduce the scope of maps of feature employed as the input information for the proceeding convolution^14^. The present method got returned; therefore, each deep feature is achieved again.

A categorizer has generated a specification based on the attributes following the present processes. Convolution procedures are utilized to extract attributes from existing structures, and a fully linked network serves as a categorizer for the present attributes. The output layer of SoftMax can be achieved by using the totally linked component for categorization purposes.

Numerous eminent methods have been developed utilizing the existing layers, including AlexNet, GoogleNet, and Xception. The organizations’ overfitting is a considerable issue during training the network. Data augmentation and layer of dropout have been found to be several approaches made for avoiding overfitting.

#### Convolutional layer

By using a layer of convolution, it is possible to apply complex operations to the input image. Several filters that have fixed-size are structured in a specific configuration to form the present layer. Local filters are incorporated in the current procedure, which results in the combination of their trained weights and biases within the picture. This technique is popularly known as weight-sharing, and it ensures that the same characteristic is reflected throughout the information.

Neuron’s local receptive region is a region the neuron is linked to it before. The filter’s dimension determines the receptive region’s dimension. The the image representation along with filters' weight and bias determine the extent of the input data and kernel. The output can be computed using either ReLu or sigmoid as the activation function.

#### Pooling layer

Convolution and activation functions were used on feature maps before pooling was carried out. The present process resulted in small attribute maps, which contain short representations of input features. Next, the operation chosen employed via transferring a window in each datum^15^. Maximum, L2 pooling procedures, and average have been found to be some operations that have been extensively employed within pooling. Pooling average result is computed by taking the average of input values. Whereas, the pooling’s maximum results are computed by increasing the pooling results to the most. Pooling operations provide two benefits, namely reducing image size and extracting independent visual elements.

#### Completely linked layer

After pooling and convoluting the data, it is condensed into a vector, which is a one-dimensional representation. This vector is fed as input to the fully connected layer. It is possible that there are hidden levels within the fully integrated system. Neurons introduce a bias value to the connected weights, which they multiply by the data existing in the prior layer. The activation function is applied to the given value before it proceeds to the next layer. This process determines the class of the value.

#### Xception model

Deep learning is an advanced type of artificial intelligence that utilizes multi-layered neural networks to acquire knowledge from extensive data, empowering the machine to make intricate decisions^16^.

The Xception network was introduced as kind of CNN (Convolutional Neural Network) in 2017^17^. The initial name for it was “Extreme Inception”. Xception has 36 convolutional layers. It consists of three steps. The first step is the entry, where there are recognizable convolutions, convolutions, and layers of convolution^18^. The second stage is the middle that possesses various convolutional layers. In addition, the number of the times that the flaw of middle is repeated is eight.

The third stage is the exit. This has been found to be ultimate motion that prepares the results that enjoys dense layer. The Xception algorithm was chosen because of its high efficiency and great outcomes in former studies. As a result, it was utilized to predict the RUL of the PEMFC. The methodology of Xception, which is a deep Convolutional Neural Network, has employed a dynamic search space reduction algorithm within the present study. The values of the dynamic search space reduction algorithm were used to create a deep network of Xception. In order for network can predict with high accuracy, the current deep network has been tested and trained via the dataset.

#### Optimizing the network

Optimizing the architecture of the Xception network is a complicated task that comprises selecting the most efficacious integration of design options and hyperparameters. To evaluate the efficiency of the network in particular tasks, comprising loss, throughput, or accuracy, a specific performance index is necessary. Each decision variable is determined by using metaheuristics, which define the kind and range of values for both the design choices and hyperparameters, as well as the searching technique and upgrading them.

Here, the chief purpose is gaining the greater accuracy of categorization on a precise dataset. By computing the loss of cross-entropy between actual labels and forecasted possibilities, it has been assessed. The cost function is calculated in the following way:

4 $$f\left(dv\right)={w}_{1}\times accuracy\left(dv\right)-{w}_{2}\times OPS(dv)$$

The decision variables of the Xception that includes selections of architecture have been illustrated by $dv$. The network’s resultant categorization accuracy has been illustrated by $accuracy(dv)$, and the expected quantity of operations that is necessary for evaluating the network has been depicted by $OPS(dv)$. The weights ${w}_{2}$ and ${w}_{1}$ specify the equilibrium between efficiency and accuracy in accordance with particular limitation and necessities.

For approximation of $OPS(dv)$, an alternative metric is utilized such as the quantity of MAC (Multiply-Accumulate. The fundamental building blocks of convolutional neural networks are the convolutional layers' stride values, kernel sizes, output and input sizes that determine the MAC procedures. Optimizing the model requires using four decision variables, including value of stride ($stride$), size of kernel (${k}_{size}$), the numeral of modules (${n}_{mod}$), and numeral of filters in each module (${n}_{filters}$). The present parameters might be illustrated as $x=\left[ {n}_{mod},{n}_{filters}, stride,{k}_{size}\right]$.

The extended expanse of the structure area and the great calculation resources at disposal elegantly bind the values of the parameters. The present worthy aim is served by the meticulously founded ranges with exact precision.

The quantity of filters that have been allotted for each module within a network has been found to be vital, which is between 16 and 1024. The great quantity of filters considerably increases illustration power of the model and it results in a larger cost of calculation and a higher overfitting risk. Additionally, larger quantity of filters might lead to decrease in spatial scopes that might have an influence on whole efficacy of the model.

For achieving optimum outcomes, the number of modules existing within a model must be taken into account that is between 1 and 10. Enhancing the quantity of modules might improve illustration power of the model; however, it accompanies costs of calculation and greater overfitting risk.

The size of kernel, which is between 1 and 7, has a vital role in specifying quantity of being granular and receptive area. A regularly employed method is using a kernel that its size is three as it endeavors to strike an equilibrium between the two aforementioned factors.

The flow of data and resolution within the network are significantly influenced by the stride value, which varies from 1 to 2. If larger stride values are used, the feature maps are reduced, but if smaller values are used, they are preserved. For layers of convolution and down-sampling, the stride values of 1 and 2 are, in turn, employed.

### Improved Chaos African Vulture Optimization algorithm

A considerable subject within numerous regions, including technical regions and engineering, is to find the minimum and maximum amount by generating the present procedure. The procedure of optimization involves choosing the most optimum solution from a range of options based on established standards. This process is used to maximize or minimize a function for a practical problem^19^.

There exist distinct optimization procedures; however, optimization algorithm might be appropriate to solve one issue might not be appropriate for another issue. A procedure’s compatibility of an issue has been affected via myriad features, including concavity and derivability of the function. The metaheuristic algorithms suggest the best results for non-linear and intricate issues among distinct kinds of optimizers. Because the present procedures do not employ differentiation, they get easier and quicker results^20^. Moreover, stochastic nature of the present procedures provides some opportunities form to be a productive and light procedures for gaining the greatest mark fir each kind of issue.

The bio-string approach regularly duplicates distinct incidences from the physics rules and nature for achieving productive optimality clarification. As a result, a quantity of types of the present procedures have been continuously represented regularly.

#### African Vulture Optimization (AVO)

The AVO is an optimizer that has been inspired by nature and hunting behavior of the vultures. A certain type of large bird exists that cannot be sterilized. However, they typically prey on sick or injured animals. Another feature of this animal is unable to tear the corpse, the search for a strong individual to do it. Moreover, when they get exhausted, the weaker individuals consume the rest. The animals are known for their exceptional strength and capability to attain great heights. These individuals are always on the move, flying from one location to another in search of the most nutritious food. Additionally, they engage in constant competition with other vultures to consume the discovered food.

Like other bio-inspired approaches, the present algorithm begins with an animal that has been selected in a random manner. As a result, power of each animal has been determined by employing it with a random amount in the assessed value of cost. After that, the best animals in the groups are evaluated and maintained. The present procedure has been explained by the proceeding equation:

5 $${R}_{i}=\left\{\begin{array}{ll}Best\; vulture \;1, & \quad   if \; {p}_{i}={m}_{1}\\ Best \; vulture \; 2, & \quad  if \; {p}_{i}={m}_{2}\end{array}\right.$$

6 $${m}_{1}+{m}_{2}=1$$

here, ${m}_{2}$ and ${m}_{1}$ have been computed prior to optimization, which are in the range of 0 and 1.

The purpose of the *Roulette wheel* is selecting the best animals of the groups.

7 $${p}_{i}=\frac{{L}_{i}}{{\sum }_{j=1}^{m}{L}_{i}}$$

here, the animals’ rate of satisfaction has been illustrated by $L$.

Consequently, the animals’ rate of starvation has been achieved. The animals go to taller peaks to discover sources of nutrition. When they do not have adequate power to hunt, they begin a battle on the issue of being with strong animals have access to nutrition. The present description can be calculated in the subsequent manner:

8 $$t=z\times \left({\text{sin}}^{w}\left(\pi \times 0.5\times \frac{{iter}_{i}}{{\text{max}}_{iter} }\right)+\text{cos}\left(\pi \times 0.5\times \frac{{iter}_{i}}{{\text{max}}_{iter} }\right)-1\right)$$

9 $$L=\left(2\times {\beta }_{1}+1\right)\times V\times \left(1-\frac{{iter}_{i}}{{\text{max}}_{iter} }\right)+t$$

here, the present iteration has been illustrated by $ite{r}_{i}$, a stochastic amount that is in the range of 1 and 0 has been demonstrated by ${\beta }_{1}$, and the constant quantity has been depicted by $w$, which is employed for determining optimization’s operation and defining stages of operation and analysis. The total number of iterations has been indicated by ${\text{max}}_{iter}$, and $V$ has been located that is chosen on the basis of stochastic manner in the range of 1 and 0. Moreover, a stochastic number has been illustrated by $z$ that is in the range of 2 and -2. In the case that $V$ reaches zero, the animal will be hungry. On the other hand, if $V$ increases to one, the animal will be able to eat.

In the end, myriad stochastic portions have been evaluated by two techniques for designing examination term of the algorithm. The nutrition hunting path has been explained by the animals on the basis of the subsequent equation:

If $${P}_{1}\ge ran{d}_{{P}_{1}}$$:

10 $$P\left(i+1\right)={R}_{i}-D\left(i\right)\times L$$

If $${P}_{1}<ran{d}_{{P}_{1}}$$:

11 $$P\left(i+1\right)={R}_{i}-L+{\beta }_{2}\times \left(\left(ub-lb\right)\times {\beta }_{3}+lb\right)$$

where,

12 $$D\left(i\right)=\left|X\times R\left(i\right)-P\left(i\right)\right|$$

The value of $X$ determines the stochastic variation in the amount of food that an animal will preserve from other animals in the team. This saved nutrition has been determined $X=2\times \beta$, two stochastic dimensions have been expressed ${\beta }_{i} (i=\text{1,2},3)$, which are between 1 and 0. The best animal has been illustrated by $R$, and the highest and lowest limitation of the parameters have been demonstrated by $ub$ and $lb$.

The other term of the present algorithm has been found to be utilization that is fulfilled once $\left|H\right|<1$. It includes two potions that has two strategies, namely rotating and siege flight. The present strategies have been computed by ${P}_{3}$ and ${P}_{2}$ as two variables that are in the range of 1 and 0. The primary section of the utilization begins once the quantity of $\left|H\right|$ is in the range of 1 and 0.5. Once $\left|H\right|\ge 1$, it depicts that these animals are fulfilled. Then, the weaker animal endeavors to obtain nutrition from the strong animal that has been displayed subsequently:

13 $$P\left(i+1\right)=D\left(i\right)\times \left(L+{\beta }_{4}\right)-d\left(t\right)$$

14 $$d\left(t\right)={R}_{i}-P\left(i\right)$$

here, ${\beta }_{4}$ indicates a stochastic quantity that is between 0 and 1.

In addition, the animal’s curved movement has been explained by the subsequent formula:

15 $${F}_{1}=R\left(i\right)\times \left(\frac{{\beta }_{5}\times P\left(i\right)\times 0.5}{\pi }\right)\times \text{cos}\left(P\left(i\right)\right)$$

16 $${F}_{2}=R\left(i\right)\times \left(\frac{{\beta }_{6}\times P\left(i\right)\times 0.5}{\pi }\right)\times \text{sin}\left(P\left(i\right)\right)$$

17 $$P\left(i+1\right)={R}_{i}-\left({F}_{1}+{\text{F}}_{2}\right)$$

here, two stochastic quantities that are in the range of 1 and 0 have been depicted by ${\beta }_{6}$ and ${\beta }_{5}$. Once $\left|H\right|$ is bigger or equivalent to 0.5, the movement of vultures attracts various individual to the source of nutrition if the quantity of $\left|H\right|$ is smaller than 0.5. Moreover, antagonistic and blockade battle have been employed for discovering the nutrition. Initially, a dimension on the basis of stochastic choice has been illustrated by ${\beta }_{{P}_{3}}$ that is between 1 and 0. Once, ${\beta }_{{P}_{3}}\ge {P}_{3}$, myriad animals fight for gaining nutrition. Once, ${\beta }_{{P}_{3}}<{P}_{3}$, the strong siege-battle technique has been employed. Then, the animals get starved that make a competition among al the animals to achieve food. It can be demonstrated by the following equation:

18 $${A}_{1}=BestVultur{e}_{1}\left(i\right)-\frac{BestVultur{e}_{1}\left(i\right)\times P\left(i\right)}{BestVultur{e}_{1}\left(i\right)-{P(i)}^{2}}\times L$$

19 $${A}_{2}=BestVultur{e}_{2}\left(i\right)-\frac{BestVultur{e}_{2}\left(i\right)\times P\left(i\right)}{BestVultur{e}_{2}\left(i\right)-{P(i)}^{2}}\times L$$

here, the most incredible animals within the two groups have been demonstrated by $BestVultur{e}_{2}\left(i\right)$ and $BestVultur{e}_{1}\left(i\right)$. Additionally, the current vector’s location of the animal has been depicted by $P(i)$:

20 $$P\left(i+1\right)=\frac{{A}_{1}+{A}_{2}}{2}$$

Once, $|L|<0.5$, previous strong animals lose their strength and obtain their ability to be in the vicinity of other animals. At the same time, each animal flies in different directions for gaining food.

21 $$P\left(i+1\right)=R\left(i\right)-\left|d\left(t\right)\right|\times L\times LF\left(d\right)$$

where, the Levy flight is indicated by $LF$ that might be assessed via the subsequent formula:

22 $$LF\left(x\right)=\frac{u\times \sigma }{100\times {\left|v\right|}^{2}}$$

23 $$\sigma ={\left(\frac{\Gamma \left(1+\beta \right)\times \text{sin}\left(\frac{\pi \beta }{2}\right)}{\Gamma \left(1+{\beta }_{2}\right)\times \beta \times 2\left(\frac{\beta -1}{2}\right)}\right)}^{\frac{1}{\beta }}$$

here, a fixed quantity has been demonstrated by $\beta$; additionally, stochastic quantities have been illustrated by $v$ and $u$ that are between 1 and 0.

#### Improved Chaos African Vulture Optimization (ICAVO)

The primary stage includes the conduction of the chaos map approach that employs unpredicted chaotic parameters rather than stochastic parameters. Chaos sequences, that have been found to be existing within non-linear and dynamic systems, are non-periodic, non-convergent, and bounded. As a result, the carry out simple explorations quicker in comparison with probability-based stochastic discovering^21^. By integrating chaotic variables within metaheuristic methods, the search space might be efficiently discovered because of the turbulence sequences’ dynamic nature. In brief, employing the chaotic parameters within the chaos map enhances the discovery of the search space by the way it permits for quicker and more effective discoveries.

Optimization approaches can use various alternative chaos maps to produce different sequences by enhancing the starting situations. The current study uses a sinusoidal chaotic map function to improve the AVO algorithm’s convergence rate. It achieves this by balancing the exploitation and exploration phases, which enables the solution space to be explored more efficiently and prevent local optima. Substituting the former $r$ random value with random values from the Chaos function is how the chaos map is implemented in the AVO algorithm. The AVO algorithm incorporates the sinusoidal chaotic map function to explore the solution space, which improves its performance by avoiding local optima. As a whole, incorporating the chaotic map function into the AVO algorithm enhances its performance by improving the solution space’s exploration and avoiding local optima. The statistical sinusoidal map formula can be utilized to analyze the ${\beta }_{i}$. Applying this equation results in the following formula:

24 $${\beta }_{i}^{t+1}=\gamma \times {\left({\beta }_{i}^{t}\right)}^{2}\text{sin}(\pi {\beta }_{i}^{t})$$

Here, the random amount produced in the present iteration is defined by ${\beta }_{i}^{t}$. A changeable variable $\gamma$ equals $2.3$. The variable ${\beta }_{i}^{0}$ is attained by trials and errors and equals $0.5$.

The algorithm undergoes another modification that is on the basis of the OBL procedure. The OBL process is a calculated instrument that orders the bio-inspired processes for adaptation. It generates various locations for the candidate’s explanations for a specific task. These new locations for individuals may lead to favorable outcomes for the cost value. It is crucial to present an enhanced explanation, quickly evaluate a candidate explanation, and connect coherent diverse explanations, in order to select the best explanation as a potential individual for the future. For candidate explanation, with name ${Y}_{i}$, the different explanation (${Y}_{i}^{{\prime}}$) is calculated in the subsequent equation:

25 $$\begin{aligned}{Y}_{i}^{\prime} & =a+b-{Y}_{i} \\ {Y}_{i} &=\in \left[\underline{y},\overline{y}\right]\end{aligned}$$

Here, the explanation space’s maximum boundary is indicated by $\overline{y}$ and its minimum boundary is indicated by $\underline{y}$.

#### Mutation of Gaussian (GM)

The bio-string methods have undergone further modifications by the GM. These modifications are inspired by the normal distribution of Gaussian. It is highly probable that Gaussian mutation is a new generation that is closely related to the core parent due to their thin tail. Consequently, the pursuit formula offers smaller phases in explaining the space’s total ways of making the best tour. This results in the possibility of offering a relatively isotropic with a greater velocity. The Gaussian’s density function can be computed using the subsequent equation:

26 $${f}_{gaussian\left(0,{\beta }^{2}\right)}\left(\alpha \right)=\frac{1}{\sqrt{2\pi {\beta }^{2}}}\text{exp}\left(-\frac{{\alpha }^{2}}{2{\beta }^{2}}\right)$$

Here, the quantity of the Gaussian is demonstrated by $a$ that is a random number between 0 and 1. The variance of the candidates is represented by ${\beta }^{2}$.

The final formula is decreased to produce a singular n-dimensional stochastic variable with a standard deviation of one and a mean of zero. This stochastic variable is then applied to the bio-string procedure equation utilizing the following formula:

27 $${Y}_{i}^{d}={Y}_{i} \odot G\left(\alpha \right)$$

Here, a Gaussian probability distribution is denoted by a Gaussian density vector $\left(\alpha \right)$.

It is possible to observe the utilization of this concept, which aims to bring renewal to the AVO. This observation reveals that:

28 $$P\left(i+1\right)=R\left(i\right) \odot G\left(\alpha \right)-\left|d\left(t\right)\right|\times L\times LF\left(d\right)$$

The procedure of learning involves creating oppositional individuals by opposing the present individuals and then updating the locations of the pursuit agent. This technique effectively expands the search area.

#### Xception based on ICAVO

The chief purpose of the present application has been found to be employing the ICAVO approach for gaining the maximum probable optimality of hyperparameters and the design of the present model. The purpose of the model’s optimality is to increase its precision and efficiency in forecasting RUL information. Considering the hyperparameters is of paramount significance, because they influence the efficacy of the network greatly^22^. The optimal values of the decision variables and efficacy metrics for design selection within the Xception model have been accurately represented in Table 1.

**Table 1:** An instance for optimum value for the decision variables within the design of the Xception model gained via employing a dynamic solution space decrease algorithm.

Decision variable	Optimal value
${n}_{filters}$	405
${n}_{mod}$	2
${k}_{size}$	4
$stride$	1

Through the implementation of a state-of-the-art dynamic solution space reduction algorithm, the highest level of proficiency was attained. This method displays unparalleled creativity as it can effortlessly adjust to a wide range of distinct scenarios and data distributions. This wonderful optimizer was utilizer for optimizing the cost function that strikes an incredible parallel between efficacy and precision of the model. It was conducted with a great meticulousness for fulfilling the criteria and limitations.

The network demonstrated exceptional proficiency by achieving a great accuracy in classification that its value was 0.95 after undergoing meticulous optimization. The present optimum value endeavors to strike a parallel between difficult illustration power of the network and its cost of calculation that solidify its state.

## Simulation results

### System configuration

The hardware setup of the system comprises a 2-Intel^®^ Xeon^®^ CPU running at a speed of 2.30GHz, along with a Tesla P100-PCIE-16GB GPU. The system is furnished with 13 GB of RAM, operating at a frequency of 1.99 GHz, and possesses a 500GB hard drive.

The billiard table utilized is approximately two-thirds the size of the standard table. The balls have a diameter of 42mm. A CCD camera, positioned above the table, captures the image of the playing surface. The captured images are then transmitted to a PC for further processing. MATLAB R2020b is the programming software installed on the system, which is powered by Windows 11 OS.

### Ball detection analysis

Table 2 illustrates a comparison of the average execution time and detection rate between the proposed method and some other state of the art methods, including multi-objective recognition (MOR)^13^, local peak edges and HSV (LPE/HSV)^23^, and artificial intelligence (AI)^24^, Hough transform (Hough)^9^, Morphological/clustering (Morphoclust)^23^, Self-Training^25^ utilized in the 8-ball billiards robot vision system.

**Table 2:** Ball detection analysis.

Approach	Elapsed time (s)	Accuracy (%)
MOR	0.6	92.44
LPE/HSV	1.2	94.08
AI	1.9	95.23
Hough	0.5	93.52
Morphoclust	2.7	95.19
Self-training	0.7	95.44
Proposed method	0.5	97.81

The results in the table highlight the effectiveness, robustness, and reliability of the multi-ball detection approach introduced in this research paper. The data in Table 2 provides valuable insights into the performance metrics of the various methods employed in the robot vision system. Upon analyzing the average execution time and detection rate for each method, it is clear that the proposed detection method in the study surpasses the other techniques in terms of efficiency and accuracy.

The results indicate that the approach outlined in the paper enhances the overall effectiveness of the 8-ball billiards robot vision system by improving its capability to detect and track multiple balls simultaneously. This improved efficiency, along with a high detection rate, demonstrates the robustness and reliability of the proposed multi-ball detection approach, making it a significant contribution to the fields of robotics and computer vision.

**Figure 7:**
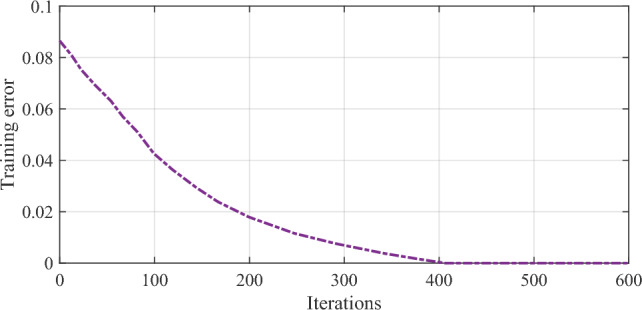
The descent curve of training error.

Therefore, the findings presented in the table emphasize the importance of the multi-ball detection method in enhancing the performance of the 8-ball billiards robot vision system, highlighting its potential for practical applications in real-world scenarios. The descent curve of training error is illustrated in Fig. 7.

After 400 iterations, the training error significantly decreased and reached a convergence point of approximately 0.0015. During the verification test of the proposed Xception/ICAVO model, both solid and striped balls achieved recognition rates surpassing 99.85%. The proposed Xception/ICAVO model will be preserved, and the weight matrices of the convolution kernels will be extracted and made ready for the subsequent CNN training process.

### Authentication

**Figure 8:**
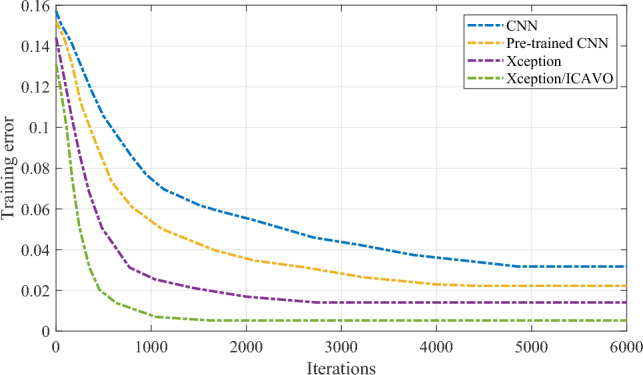
The descent curve of training error.

400 images from the dataset were utilized in training the proposed CNN to develop a ball pattern recognition classifier model. Following the training process, 100 images were set aside for test. There was no overlap between the images used for training and those used for testing. To provide a proper validation, the results of the proposed model were compared with some other similar state of the art models, including original CNN^13^, pre-trained CNN^13^, and original Xception network. The weights from a designed model were employed as the initial weights for the proposed Xception/ICAVO training, with the training error curve depicted in Fig. 8.

However, during the course of training, a noticeable divergence in performance emerges. The Xception/ICAVO model sets itself apart by showcasing a rapid and significant reduction in training error, indicating an effective learning process and a strong adaptation to the dataset. It is worth highlighting that despite starting on equal footing with the original CNN, the Xception/ICAVO model quickly surpasses it, along with the pre-trained CNN and original Xception network, in terms of learning efficiency. The graph likely illustrates the Xception/ICAVO model reaching a lower error plateau, showcasing superior performance in identifying patterns within the ball dataset. This implies that the Xception/ICAVO model, with its initial weights guided by a tailored model, is highly efficient for the given task, potentially presenting a substantial enhancement over the cutting-edge models it was pitted against. During the test, the recognition rates for solid and striped balls increased to 98.65% and 98.927% respectively.

## Discussions

The results of this study showed significant progress in the development of robotic vision systems, especially in the realm of 8-ball billiards. By combining advanced color image processing methods with a custom convolutional neural network, the issues related to object detection and ball pattern recognition in this game have been effectively addressed. The main innovation, the ICAVO algorithm, has greatly improved the Xception network, leading to better accuracy and dependability. In the upcoming section, we will delve deep into the findings, examine how they impact robotics and computer vision, and look into possible avenues for future research.

### Performance analysis

In the previous section, we showed the outstanding performance of the multi-ball detection approach compared to other cutting-edge methods. The metrics for average execution time and detection rate highlight the efficiency of our method. The training error curve for the Xception/ICAVO model also confirms its effectiveness, as it quickly converges to a low error value. This demonstrates that the model has successfully learned to identify patterns in the ball dataset.

During the verification test, the Xception/ICAVO model achieved exceptional recognition rates of over 99.85% for both solid and striped balls. This high level of accuracy proves the model's reliability in distinguishing between the two types of balls. Furthermore, by training the CNN classifier with the weight matrices extracted from the Xception/ICAVO model, we were able to further enhance the system's performance. The test results for solid and striped ball recognition were 98.65% and 98.927% respectively, which clearly demonstrate the effectiveness of our approach.

### Implications for robotics and computer vision

This research goes beyond just 8-ball billiards. Creating a high-tech robotic vision system for this sport shows that similar systems could be used in other areas too. Being able to accurately spot and follow multiple objects, like billiard balls, has many uses in different industries. For instance, robotic vision systems could change industrial automation by making it easier to recognize and handle objects precisely. Also, the findings from this research could improve surveillance systems, making it easier to spot and track multiple targets at once. By combining the ICAVO algorithm with the Xception network architecture, a stronger and more flexible system has been created. The ICAVO algorithm's efficiency in exploring solutions and avoiding local optima has greatly improved the system's overall performance. This upgrade to the Xception network shows that there's potential for even more improvements by using advanced optimization algorithms.

### Limitations and future directions

Although the research has shown promising results, there are some limitations to keep in mind. The study focused on 8-ball billiards, so the system's performance might differ with various ball arrangements or table sizes. It would be interesting to see how the system performs on a full-size billiard table and its adaptability to other billiard games like 9-ball or snooker. Moreover, incorporating more advanced image processing techniques or optimizing the CNN architecture could lead to further improvements.

Another potential area for future research could involve transfer learning. Utilizing the weight matrices from the Xception/ICAVO model as a pre-trained model for similar object recognition tasks could speed up the training process and enhance performance in related vision-based applications. Additionally, exploring the combination of deep learning techniques with robotic control systems could enable more complex robotic behaviors.

In summary, this research has advanced the development of a sophisticated robotic vision system for 8-ball billiards. By integrating color image processing, deep learning, and optimization algorithms, a highly effective system has been achieved. The implications of this work go beyond the specific application, demonstrating the potential for advanced robotic vision systems in various fields. Future research avenues include further system enhancements, transfer learning exploration, and the integration of robotic control systems with vision capabilities.

## Conclusions

The research presented in this paper represented a significant advancement in the field of robotic vision systems, specifically in the context of 8-ball billiards. Through the utilization of advanced color image processing techniques and the incorporation of a customized convolutional neural network, the study effectively addressed the challenges associated with objective detection and the identification of ball patterns. The key innovation lied in the implementation of an improved variant of the Chaos African Vulture Optimization algorithm, which played a crucial role in refining the Xception network. This refinement process greatly enhanced the precision and reliability of the system. The implications of these improvements were far-reaching. Firstly, they contributed valuable insights and methodologies to the broader field of robotics, enriching the existing knowledge base and establishing new standards for future research. Secondly, the practical applications of this research had the potential to revolutionize the automation landscape in the sport of billiards. By introducing a level of intelligence and sophistication previously unseen in sports equipment, this study offered a glimpse into the future of technology integration in sports. Moreover, the applications of such advanced robotic vision systems extended beyond the billiard table. They could be adapted to various fields that required meticulous visual recognition and classification, ranging from industrial automation to surveillance and beyond. The principles and technologies developed in this study had the potential to enhance the capabilities of machines in interpreting and interacting with their environments.

## Data Availability

All data generated or analysed during this study are included in this published article.
